# A hybrid deep neural network for classification of schizophrenia using EEG Data

**DOI:** 10.1038/s41598-021-83350-6

**Published:** 2021-02-25

**Authors:** Jie Sun, Rui Cao, Mengni Zhou, Waqar Hussain, Bin Wang, Jiayue Xue, Jie Xiang

**Affiliations:** 1 College of Information and Computer, Taiyuan University of Technology, Taiyuan, China; 2 College of Software, Taiyuan University of Technology, Taiyuan, China; 3 Graduate School of Interdisciplinary Science and Engineering in Health Systems, Okayama University, Okayama, Japan

**Keywords:** Classification and taxonomy, Computational neuroscience, Image processing, Machine learning

## Abstract

Schizophrenia is a serious mental illness that causes great harm to patients, so timely and accurate detection is essential. This study aimed to identify a better feature to represent electroencephalography (EEG) signals and improve the classification accuracy of patients with schizophrenia and healthy controls by using EEG signals. Our research method involves two steps. First, the EEG time series is preprocessed, and the extracted time-domain and frequency-domain features are transformed into a sequence of red–green–blue (RGB) images that carry spatial information. Second, we construct hybrid deep neural networks (DNNs) that combine convolution neural networks and long short-term memory to address RGB images to classify schizophrenic patients and healthy controls. The results show that the fuzzy entropy (FuzzyEn) feature is more significant than the fast Fourier transform (FFT) feature in brain topography. The deep learning (DL) method that we propose achieves an average accuracy of 99.22% with FuzzyEn and an average accuracy of 96.34% with FFT. These results show that the best effect is to extract fuzzy features as input features from EEG time series and then use a hybrid DNN for classification. Compared with the most advanced methods in this field, significant improvements have been achieved.

## Introduction

Schizophrenia is a mental disorder from which 1% of the global population suffers^1^. In a clinic, doctors directly judge schizophrenia by electroencephalography (EEG). Although this method has a certain effect, it needs a substantial amount of time and energy and is not suitable for a large number of accurate diagnoses of schizophrenia. Researchers subsequently introduced a model using a computer, which reduced the workload and accelerated the diagnostic speed of EEG.

EEG is a low-cost and noninvasive measurement network tool and an effective tool for recording brain activity ^2^. In recent years, EEG has been extensively utilized in the research and diagnosis of various nervous system diseases, including epilepsy^3^, Alzheimer’s disease (AD)^4^ and schizophrenia^5^. EEG signals show the complex information in the brain, which has a high dimension and contains a considerable amount of information, and are difficult to analyze directly. Therefore, some auxiliary means are necessary to extract useful information about the brain. Feature extraction is an effective method for studying EEG data. By extracting some useful features from a large quantity of original signals, the purpose of reducing feature dimensions is achieved, and the physical meaning of individual features is not destroyed during this process. For schizophrenia, some feature extraction methods (such as those employed for time-domain features^6^ and frequency-domain features^7^) have been proposed to quantify EEG signals for studying state changes in the brain. Currently, whether time-domain features or frequency-domain features can more effectively distinguish brain differences between patients with schizophrenia and healthy controls remains ambiguous. Therefore, one of the purposes of this study is to compare the ability of different features to extract EEG signals.

Machine learning (ML) can be used to develop computer-aided diagnostic tools for clinical applications and explore the pathophysiological mechanisms of diseases. ML has revolutionized the field of schizophrenia by providing a tool to solve the high complexity of EEG signals. In the past few years, traditional ML technology (that is, a non-deep learning (DL) algorithm) has been the only method of choice in EEG analysis and has been combined with various feature extraction methods ^8,9^. In a relatively new development, DL algorithms have been extensively applied in medical image and signal processing and have shown high research potential. In most cases, their performance exceeds traditional machine learning techniques^10^. In the process of disease diagnosis and classification, an increasing number of researchers have applied DL to the field of EEG to study mental diseases^11–14^. We performed a search on Web of Science and PubMed using the following group of keywords: “schizophrenia” AND “EEG” AND (“machine learning” OR “deep learning”). References from 2000 until 2019 were utilized for further analysis; the accuracy of these articles is reflected in Table 1. In addition, an increasing number of researchers have employed hybrid structures to design neural network structures. Specifically, the convolutional neural network (CNN) is used for learning task-related features, processing pictures, and mining interchannel correlation from frames via designed convolutional filters. Long-short-term memory (LSTM) networks are composed of recurrent networks that include memory to model temporal dependencies in time series problems. This approach gives us a way to structure our research.

**Table 1:** List of published works on schizophrenia classification using EEG signals in recent years.

Author (year)	EEG dataset	Rest/task	Sampling rate	Channels	Features	Classifier	Accuracy
Bose et al., 2016^16^	57 schizophrenia patients and 24 normal subjects	Rest	256	23	Absolute power analysis	SVM	83.33%
Johannesen et al., 2016^53^	40 schizophrenia patients and 12 healthy controls	Task	1024	60	Morlet continuous wavelet transform	SVM	87%
Jeong et al., 2017^54^	30 schizophrenia patients and 15 controls	Task	1024	14	Mean subsampling technique	SKLDA	Over 98%
Piryatinska et al., 2017^55^	45 boys suffering from schizophrenia and 39 healthy boys	Rest	128	16	є-complexity of a continuous vector function	RF	85.3%
Chu et al., 2017^56^	10 normal and 17 markedly ill schizophrenic patients	Task	256	31	ApEn	SVM	81.5%
Alimardani et al., 2018^57^	26 subjects with schizophrenia and 27 patients with BMD	Rest	250	22	DB-FFR	NN	87.51%
Alimardani et al., 2018 ^58^	23 bipolar disorder and 23 schizophrenia subjects	Rest	250	21	SSVEP SNR	KNN	91.30%
Phang et al., 2019^59^	45 schizophrenia patients and 39 healthy controls	Rest	128	16	Vector-autoregression-based directed connectivity (DC), graph-theoretical complex network (CN)	DNN-DBN	95%
Phang et al.,2019^60^	45 schizophrenia patients and 39 healthy controls	Rest	128	16	Directed connectivity measures (VAR coefficients and PDCs) and topological CN measures	MDC-CNN	91.69%
Oh et al.,2019^14^	14 healthy subjects and 14 SZ patients	Rest	250	19	**–**	CNN	98.07%
Present work	54 patients with schizophrenia and 55 healthy controls	Rest	500	60	FuzzyEn	CNN + LSTM	99.22%

In this study, we propose DL algorithms for use in the analysis of EEG signals for schizophrenia research to improve the classification accuracy. We first divide the EEG signal into three bands and extract different domain features from each band. These bands are then constructed to input red–green–blue (RGB) images into our network. Furthermore, we construct a hybrid DL network that integrates a CNN and LSTM for processing the EEG-based schizophrenia classification and obtains a high classification accuracy.

This article is organized as follows: “New method” describes the proposed method and the DL structure, and “Results” introduces the specific baseline method. The data acquisition method is presented in  “Comparison with Different Deep Learning Models”. “Discussion” describe the experimental results that are achieved and present a discussion, respectively. The conclusions are detailed in “Conclusions”.

## New method

EEG signals are nonstationary signals that contain complex brain activity information, but some of these features cannot be estimated from these signals. To preserve the continuous time relationship and internal characteristics of the EEG time series, we divide each trial into 6 time windows (each time window is 400 ms, and adjacent time windows overlap by 200 ms). The EEG signals contain multiple frequency characteristics. Based on previous research, the frequency band power of the EEG in the θ, α, and β bands of schizophrenic patients is different than that of healthy controls ^15–17^. Therefore, we divide the whole frequency spectrum into three sub-bands: theta (4–7 Hz), alpha (8–13 Hz), and beta (14–30 Hz).

### Participants

Fifty-four patients with schizophrenia [36 male patients and 18 female patients, with a mean age = (37.80 ± 1.34)] and fifty-five healthy controls [31 male controls and 24 female controls, with a mean age = 41.00 ± 1.59] were included in this study. The patients were recruited from Huilongguan Hospital in Beijing, China. The schizophrenic patients were diagnosed according to the fourth edition of the Diagnostic and Statistical Manual of Mental Disorders (DSM-IV) and World Health Organization (ICD-10, 10th revision of the International Classification of Diseases) criteria for a lifetime diagnosis of schizophrenia or schizophrenia spectrum disorder and were recruited from consecutive admissions to a psychiatric hospital. These patients had been treated with stable doses of antipsychotic medications. In addition, the healthy controls did not have any history of mental illness or drug abuse. This study complies with the Code of Ethics of the Declaration of Helsinki. The study protocol was approved by Beijing Huilongguan Hospital. All the participants provided written informed consent as approved by the institutional review board. The full name of the approving committee is Beijing Huilongguan Hospital' s ethics committee, which affirmed its approval of the study.

The characteristics of all the participants, including age, sex, illness course and age at disease onset, are shown in Table 2. Each characteristic is averaged, and the standard error (SE) is shown in parentheses. No statistically significant difference in the ages of the healthy controls and schizophrenia patients (*p* > 0.05) was obtained.

**Table 2:** Comparison of the demographic characteristics between healthy controls and schizophrenic patients.

Characteristics	Normal (n = 55)	Schizophrenia (n = 54)	t/χ2 value	P-value
Mean age (SE), years	41.00 (1.59)	37.80 (1.34)	0.464	0.597
Male/female	31/24	36/18	–	–
Mean illness course (SE), year	–	15.07 (1.22)	–	–
Mean (SE) age at disease onset	–	24.21 (0.96)	–	–

### Feature extraction

Accurately extracting EEG signal features is not only challenging but also an essential step in classification because this extraction determines the classification accuracy. The EEG signals of schizophrenia can be extracted by time-domain feature methods and frequency-domain feature methods.

Time-domain feature extraction methods study EEG signals using variations in signal time series. The complexity of EEG reflects the irregularity or unpredictability of brain activity. With the continuous advancement and development of nonlinear theory, many researchers are applying nonlinear analysis methods to EEG data analysis. Entropy is a nonlinear analysis method that can be used to measure the complexity. Entropy is the most commonly employed feature index among the time-domain features and is extensively employed in the diagnosis of diseases. Among the commonly employed entropies, fuzzy entropy (FuzzyEn) was developed based on other entropies. Compared with other entropies, such as information entropy, sample entropy and FuzzyEn have the advantages of excellent robustness and strong antinoise ability, and the algorithm complexity is lower. The entropy value measured by fuzzy entropy is continuously stable and less sensitive to the noise of EEG data, which renders it more suitable for analyzing chaotic signals. Previous studies have proven that the ability of FuzzyEn to detect and recognize signals is superior to the ability of other entropies for both epilepsy^18^ and schizophrenia^19^.

The frequency-domain feature extraction method primarily studies the EEG signal by converting the original time-domain signal into the frequency-domain signal, which reflects the relationship between the frequency and its corresponding amplitude and can mine the deeper features of signals. Among the common frequency-domain features, the Fourier transform (FT) is extensively applied. Compared with other frequency-domain features, the FT has a low computational cost and can be easily implemented. In addition, the Fourier transform requires that the signals in the frequency domain are stable EEG samples. This type of analysis is most suitable for studying EEG signals. Signals are generally continuous, but computers cannot process continuous signals, so only continuous signals are discretized. The discrete Fourier transform (DFT) reflects the discrete form of the FT in the time and frequency domains. The fast Fourier transform (FFT) is essentially a simple DFT algorithm. In previous studies, many researchers have preferred the FFT when processing EEG signals for frequency-domain features^20^.

According to the current research results in related fields, FuzzyEn and FFT are typical algorithms for time- and frequency-domain features, and both feature extraction methods have been proven to achieve excellent results and are extensively utilized in various biological signal research. In this study, to select better features to represent EEG signals, we compare the two features that are extensively applied in the time and frequency domains: FuzzyEn and FFT.

#### Fuzzy entropy

FuzzyEn is a nonlinear indicator to evaluate the occurrence probability of newly generated patterns based on fuzzy theory. In 2007, Chen et al.^21^, based on the sample entropy (SampEn) algorithm, proposed a new algorithm for measuring the complexity of time series-fuzzy entropy. By blurring the similarity measurement formula, the limitation of SampEn is eliminated^22,23^.

The FuzzyEn algorithm is described as follows:

1. For a time series of N length, the algorithm is expressed as [u(1),u(2),⋯,u(N)].
2. Carry out phase space reconstruction of the original time series and define the dimension $\mathrm{m}(\mathrm{m}\le \mathrm{N}-2)$ of phase space. After reconstruction, as shown in formula (1),
  1 $$X_{i}^{m} = \left\{ {u(1,u\left( {i + 1} \right), \ldots ,u(i + m - 1))} \right\}$$
  $i=1,2,\dots ,N-m+1$, ${U}_{0}(i)$ is the average, and the formula is shown in (2)
  2 $$U_{0} \left( i \right) = \frac{1}{m}\mathop \sum \limits_{j = 0}^{m - 1} u(i + j)$$
3. The distance ${\mathrm{d}}_{\mathrm{ij}}^{\mathrm{m}}$ is defined as the maximum difference between the corresponding elements of vector ${\mathrm{X}}_{\mathrm{i}}^{\mathrm{m}}$ and vector ${\mathrm{X}}_{\mathrm{j}}^{\mathrm{m}}$, that is,
  3 $$d_{ij}^{m} = d\left[ {X_{i}^{m} ,X_{j}^{m} } \right] = \mathop {\max }\limits_{p = 1,2, \ldots ,m} \left( {\left| {u\left( {i + p - 1} \right) - u_{0} \left( i \right)} \right| - \left| {u\left( {j + p - 1} \right) - u_{0} \left( j \right)} \right|} \right)\quad (i,j = 1,2, \ldots ,N - m + 1,j \ne i$$
4. The similarity between vector ${\mathrm{X}}_{\mathrm{i}}^{\mathrm{m}}$ and vector ${\mathrm{X}}_{\mathrm{j}}^{\mathrm{m}}$ is defined by the fuzzy membership function $({\mathrm{d}}_{\mathrm{ij}}^{\mathrm{m}},\mathrm{n},\mathrm{r})$, as shown in formula (4):
  4 $$D_{ij}^{m} = \mu \left( {d_{ij}^{m} ,n,r} \right) = exp\left( { - \frac{{\left( {d_{ij}^{m} } \right)^{n} }}{r}} \right)$$
  $\upmu \left({\mathrm{d}}_{\mathrm{ij}}^{\mathrm{m}},\mathrm{n},\mathrm{r}\right)$ are exponential functions; n and r are the gradient and the width, respectively, of the exponential functions.
5. ${\mathrm{\varnothing }}^{\mathrm{m}}\left(\mathrm{n},\mathrm{r}\right)$ is shown in formula (5):
  5 $$\emptyset^{m} \left( {n,r} \right) = \frac{1}{N - m}\mathop \sum \limits_{i = 1}^{N - m} \left[ {\frac{1}{N - m - 1}\mathop \sum \limits_{j = 1,j \ne i}^{N - m} D_{ij}^{m} } \right]$$
6. By adding dimension m + 1, the ${\mathrm{\varnothing }}^{\mathrm{m}+1}\left(\mathrm{n},\mathrm{r}\right)$ function is obtained:
  6 $$\emptyset^{m + 1} \left( {n,r} \right) = \frac{1}{N - m}\mathop \sum \limits_{i = 1}^{N - m} \left[ {\frac{1}{N - m - 1}\mathop \sum \limits_{j = 1,j \ne i}^{N - m} D_{ij}^{m + 1} } \right]$$
7. The FuzzyEn is
  7 $$FuzzyEn\left( {m,n,r} \right) = \mathop {lim}\limits_{N \to \infty } \left[ {ln \Phi^{m} \left( {n,r} \right) - ln \Phi^{m + 1} \left( {n,r} \right)} \right]$$
8. However, the length of the time series N is limited in the actual operation, and the FuzzyEn is estimated as follows:
  8 $$FuzzyEn\left( {m,r,N} \right) = ln \Phi^{m} \left( {n,r} \right) - ln \Phi^{m + 1} \left( {n,r} \right)$$

The similarity tolerance limit r and the dimension m of the phase space reconstruction parameters are the main parameters in this algorithm, and r represents the width of the boundary of the exponential function in practical application. In general, the larger is the r value, the greater is the amount of information that is lost, the smaller is the r value, and the more sensitive the result to noise will be. The r value is usually 0.1–0.25 times the standard deviation (SD) of the original time series, while the m value is usually 1 or 2. The parameters r = 0.25 and m = 2 are utilized in this study.

#### Fast Fourier transform

The FFT algorithm is an improved version of the DFT algorithm that involves fast implementation of the DFT method^24^. According to previous research ^25^, the DFT execution time is higher than the FFT execution time. Compared with the DFT algorithm, the FFT algorithm can obtain faster results when analyzing EEG signals. In previous studies, the FFT algorithm has been employed as a frequency-domain signal to extract features of various neurological disorders, including epileptic seizures^26^ and AD^27^.

The FFT algorithm is described as follows:

1. The number of sequence points is $\mathrm{N}={2}^{\mathrm{M}}$, and M is an integer; then, the DFT of $\mathrm{x}(\mathrm{n})$ is expressed as follows:
  9 $$X\left( k \right) = \mathop \sum \limits_{n = 0}^{N - 1} x(n)e^{ - j2\pi nk/N} = \mathop \sum \limits_{n = 0}^{N - 1} x\left( n \right)W_{N}^{nk} ,\quad 0 \le k \le N - 1.$$
2. The DFT operation of N points is decomposed into two groups of DFT operations of $\mathrm{N}/2$ points, that is, $\mathrm{x}(\mathrm{n})$ is decomposed into two groups: the first group is the even term; the second group is the odd term. The decomposition process is shown in formula (10):
  10 $$\begin{aligned} X\left( k \right) & = \mathop \sum \limits_{n = 0}^{N/2 - 1} x\left( n \right)W_{N}^{nk} + \mathop \sum \limits_{{n = \frac{N}{2}}}^{N - 1} x\left( n \right)W_{N}^{nk} \\ & = \mathop \sum \limits_{n = 0}^{N/2 - 1} x\left( n \right)W_{N}^{nk} + \mathop \sum \limits_{n = 0}^{N/2 - 1} x\left( {n + N/2} \right)W_{N}^{{\left( {n + N/2} \right)k}} \\ & = \mathop \sum \limits_{n = 0}^{N/2 - 1} \left[ {x\left( n \right) + W_{N}^{nk/2} x\left( {n + N/2} \right)} \right]W_{N}^{nk} \\ & \quad (W_{N}^{nk/2} = ( - 1)^{k} ,\;\;\;k = 0,1, \ldots ,N - 1) \\ \end{aligned}$$
3. Decompose $\mathrm{x}(\mathrm{k})$ into even and odd groups.
  If k takes an even number, when $\mathrm{k}=2\mathrm{r},\mathrm{r}=\mathrm{0,1},2,\dots ,\mathrm{N}/2-1$,
  11 $$X\left( {2r} \right) = \mathop \sum \limits_{n = 0}^{N/2 - 1} \left[ {x\left( n \right) + x\left( {n + \frac{N}{2}} \right)} \right]W_{N/2}^{nk}$$
  If k takes an odd number, when $\mathrm{k}=2\mathrm{r}+1,\mathrm{r}=\mathrm{0,1},2,\dots ,\mathrm{N}/2-1$,
  12 $$X\left( {2r + 1} \right) = \mathop \sum \limits_{n = 0}^{N/2 - 1} \left[ {x\left( n \right) - x\left( {n + \frac{N}{2}} \right)} \right]W_{N/2}^{nr} W_{N}^{n}$$
4. If $${\text{g}}\left({\text{n}}\right)= \text{x} \left({\text{n}}\right)\text{+x(n+N/2)}$$
  $$h\left( n \right) = \left[ {x\left( n \right) - x(n + N/2)} \right]W_{N}^{n} ,\;\;n = 0,1,2 \ldots ,\frac{N}{2} - 1,\;\;n = 0,1, \ldots \frac{N}{2} - 1$$
  13 $$X\left( {2r} \right) = \mathop \sum \limits_{n = 0}^{N/2 - 1} g\left( n \right)W_{N/2}^{nr}$$
  14 $$X\left( {2r + 1} \right) = \mathop \sum \limits_{n = 0}^{N/2 - 1} h\left( n \right)W_{N/2}^{nr}$$

Based on these calculations, the DFT of one N point can be decomposed into two DFTs according to parity because the previously mentioned N is even, and the DFT can be further decomposed to the first decomposition. The DFT of one N point can also be decomposed into two arrays according to parity. From 1 to 2, and from 2 to 4… According to this rule, the DFT can be decomposed M times into two points of addition and subtraction. This kind of process forms the butterfly algorithm of the FT. In this study, the FFT uses 200 sampling points, and the EEG data frequency is 500 Hz.

### Making images

In the previous section, we introduced the features of FuzzyEn and FFT for different applications. The standard approach in EEG data analysis is to form a feature vector of all electrodes. However, this approach disregards the spatial, spectral, and temporal structure of the data. Conversely, to maintain the spatial structure, we recommend converting the measurement results into a two-dimensional (2D) image and using multiple color channels to represent the spectral dimension.

**Figure 1:**
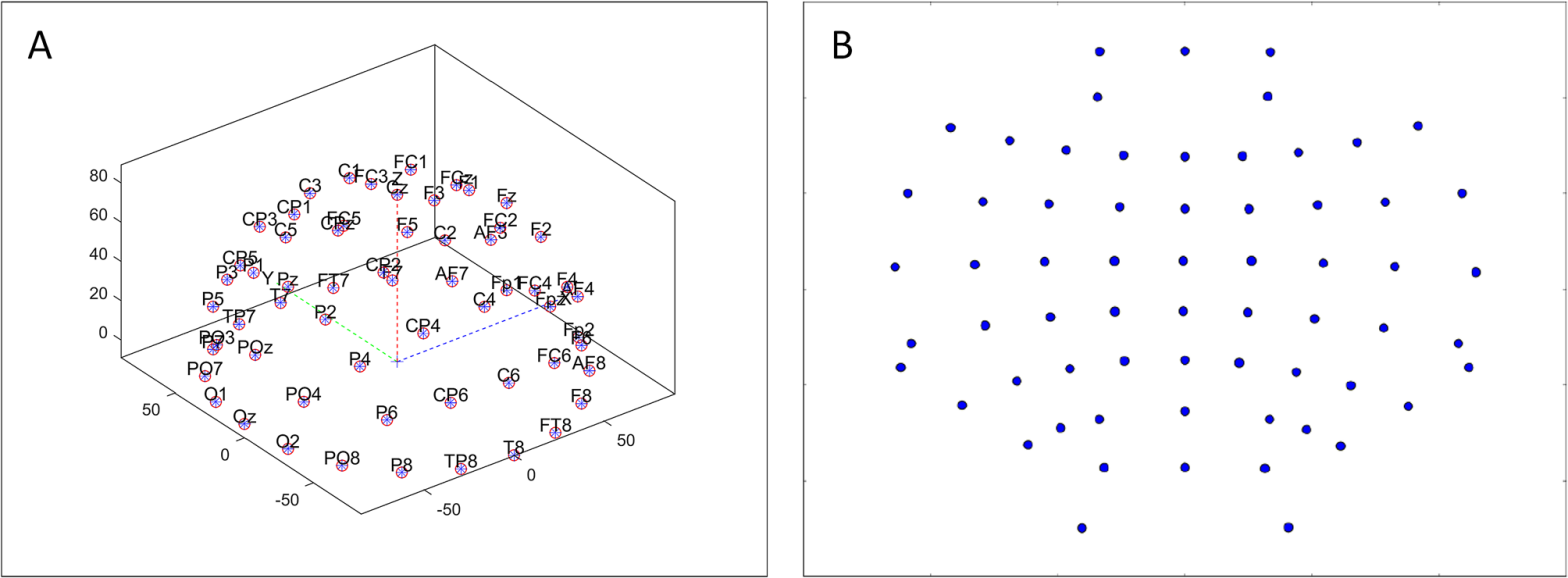
Projection of the electrode positions. (**A**) Locations of the electrodes in the original 3D space; (**B**) 2D projection of the electrode locations using the AEP.

The EEG electrodes are distributed in the three-dimensional space of the cerebral cortex sphere. To convert the spatial distribution of the electrodes into a 2D image and maintain the relative distance between adjacent electrodes, we project the electrodes from the position in three-dimensional space onto the two-dimensional surface. To ensure that the distance between all points and the center point is proportional, we use the azimuthal equidistant projection (AEP) in surveying and mapping applications^28^. In our example, the sphere can be applied to approximate the shape of the head covering or it can be used to calculate the projection on the two-dimensional surface, where the head apex is tangent to the electrode position. In addition, the use of isometric projection methods helps to interpret image and feature map visualization data and classify cognitive load levels better than standard nonspatial methods^29^. When applying AEP to the three-dimensional electrode position, we obtained the two-dimensional projection position of the electrode (Fig. 1). The spatial distribution of cortical activity is represented by the width and height of the image. We apply the Clough-Tocher scheme^30^ to interpolate the scattered power measurements over the scalp and estimate the values between the electrodes over a 32 × 32 mesh. The choice of 32 for the image size is a trade-off between the signal resolution and the computational cost.

**Figure 2:**
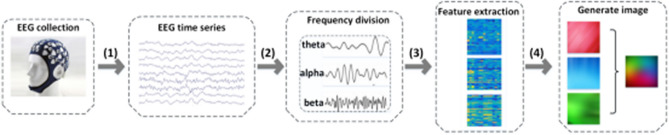
Process of generating RGB images. (1) EEG time series values from multiple locations are acquired; (2) EEG time series is divided into three subbands: theta, alpha and beta; (3) features are extracted for the three prominent frequency bands; (4) topographical maps are formed for each feature, and the sequences of topographical maps are combined to form a sequence of 3-channel images.

Figure 2 illustrates the process of generating RGB images from the EEG data. First, we divide the EEG time series into three sub-bands (theta, alpha and beta) and calculate the FuzzyEn or FFT values for the three frequency bands. Second, the AEP process is repeated for the theta, alpha, and beta bands to produce three topographical activity maps, which are then merged to form an image with three (color) channels. Last, these three (color) channels are fed into the hybrid DNN for representation learning and classification.

### Construction of the hybrid DNN

**Figure 3:**
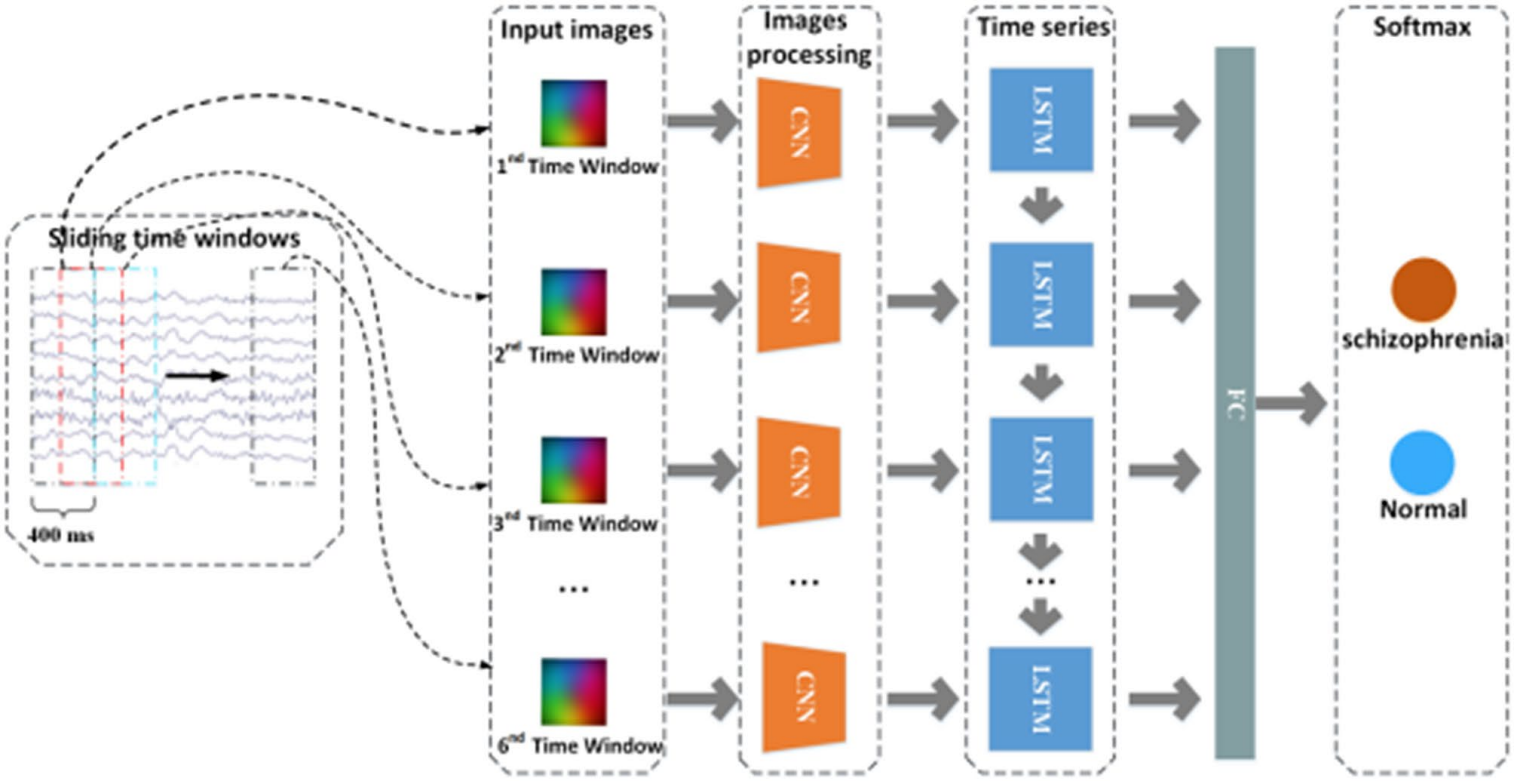
Structure of the hybrid DNNs. The EEG time series generates 6 RGB images. The images enter the CNN and then enter the LSTM layer, followed by the fully connected layer. The classification is carried out by the SF layer.

We propose a model based on hybrid DL to distinguish schizophrenic patients from healthy controls (Fig. 3). First, we divided each experiment into 6 time windows. In this way, a single RGB image can be constructed from the EEG signal in a time window. When the time window slides, an RGB image sequence can be obtained from the EEG signals. Second, we construct the hybrid DNN, which includes two types of DL structures: a CNN and an LSTM unit. The CNN unit processes images and extracts features from the RGB images. The LSTM unit is an improved recurrent neural network (RNN) structure that models context information with long-term sequences of arbitrary length. Third, the useful information obtained by the network is entered into the fully connected (FC) layer. Last, the model ends with a softmax (SF) layer to achieve the binary classification result (i.e., schizophrenia or normal).

#### CNN

CNNs are a subset of DL networks that have received widespread attention in recent years and are often applied for image recognition. The CNN architecture consists of three types of layers: (1) convolutional, (2) pooling, and (3) FC layers ^31^.

1. Convolutional layer: The convolutional layer is composed of a filter (kernel), which passes the EEG image and outputs a feature map. The convolution operation is expressed as
  15 $$y = \frac{n + 2p - f}{s} + 1$$
  where n, p, f and s denote the matrix of the input picture, the padding size, the matrix of the filter, and the stride, respectively. The size of the output matrix is $y\times y$. Next, the activation function follows the convolution layer and provides a nonlinear attribute structure, which gives the DNN the learning ability to conduct hierarchical nonlinear mapping. The most commonly employed activation function in CNNs is referred to as a rectified linear unit (ReLU, $f\left(X\right)=max(0,x)$), which provides better performance in terms of generalization and learning time.
2. Pooling layer: The pooling layer decreases the size of the feature map by the average (average pooling) or maximum (max pooling) while preserving the significant features, which reduces the computational intensity and prevents overfitting. The size of the output matrix is $y\times y$,
  16 $$y = \frac{n - f}{s} + 1$$
  where* n* denotes the matrix of the input picture, f is the matrix of the filter, and s is the number of strides.
3. FC layer: The convolution layer (+ ReLU) and pooling layers are followed by one or more FC layers. The following equation is used to connect each neuron in this structural layer with each neuron in the next layer:
  17 $$y_{j} = \sum w_{j} *x_{i} + b_{j}$$
  where w and b represent the weight and the deviation, respectively; x represents the output of the previous layer; y represents the output of the current layer; i represents the previous layer; and j represents the current layer. The output of the last FC layer is input into the SF function, and the class is predicted by determining the probability that each EEG signal indicates a normal person or a schizophrenic patient.where w and b represent the weight and the deviation, respectively; x represents the output of the previous layer; y represents the output of the current layer; i represents the previous layer; and j represents the current layer. The output of the last FC layer is input into the SF function, and the class is predicted by determining the probability that each EEG signal indicates a normal person or a schizophrenic patient.

We compare four CNN models with different depths and configurations in Table 3. The convolutional layer parameters are denoted by Conv < Layer i (1, 2, and 3) >—< number of kernels > , where Layer i (1, 2, 3) represents the ith convolutional layer. CNN structure A only involves two convolutional layers (Conv1–32) that are superimposed, followed by a max pooling layer (Max-pooling1). Compared to configuration A, configuration B adds two more convolutional layers (Conv2–64), which are followed by another max pooling layer (Max-pooling2). Compared to configuration B, configuration C adds a convolutional layer (Conv3–128), followed by another maximum pool layer (Max-pooling3). Compared to configuration C, Configuration D starts with 4 layers of Conv1–32 convolutional layers instead of 2 layers. An FC layer with 512 nodes (FC-512) is added to the architecture.

**Table 3:** Configuration information of different CNN models. The convolutional layer parameters are denoted as Conv < Layer i (1, 2, 3) >—< number of kernels > .

A < 2,0,0 >	B < 2,2,0 >	C < 2,2,1 >	D < 4,2,1 >
Input ($32\times 32$ 3-channel EEG data)			
			Conv1–32
Conv1–32	Conv1–32	Conv1–32	Conv1–32
Conv1–32	Conv1–32	Conv1–32	Conv1–32
			Conv1–32
Max-pooling1			
	Conv2–64	Conv2–64	Conv2–64
	Conv2–64	Conv2–64	Conv2–64
	Max-pooling2		
		Conv3–128	Conv3–128
		Max-pooling3	
FC-512			

We adopt an architecture that mimics a visual geometry group (VGG) network that is used in image classification tasks^32^. In our research, the input three color channels of the RGB image size of the networks are $32\times 32$ pixels. All the convolutional layers use small $3\times 3$ receptive fields, a stride of 1 pixel, a padding of 1 pixel and an ReLU activation function. This work uses the largest pool for subsequent operations. The maximum value in each feature map is selected to reduce the number of output neurons, which is a process that is performed over a $2\times 2$ window with a stride of 2 pixels. The main parameters of the CNN model are listed in Table 4.

**Table 4:** Main parameters of the CNN model.

Layer	Filter size	Number of filters	Number of neurons	Stride	Padding
Conv1	3 $\times$ 3	32	–	1	1
Max-pooling 1	2 $\times$ 2	–	–	2	0
Conv2	3 $\times$ 3	64	–	1	1
Max-pooling 2	2 $\times$ 2	–	–	2	0
Conv3	3 $\times$ 3	128	–	1	1
Max-pooling 3	2 $\times$ 2	–	–	2	0

#### LSTM

**Figure 4:**
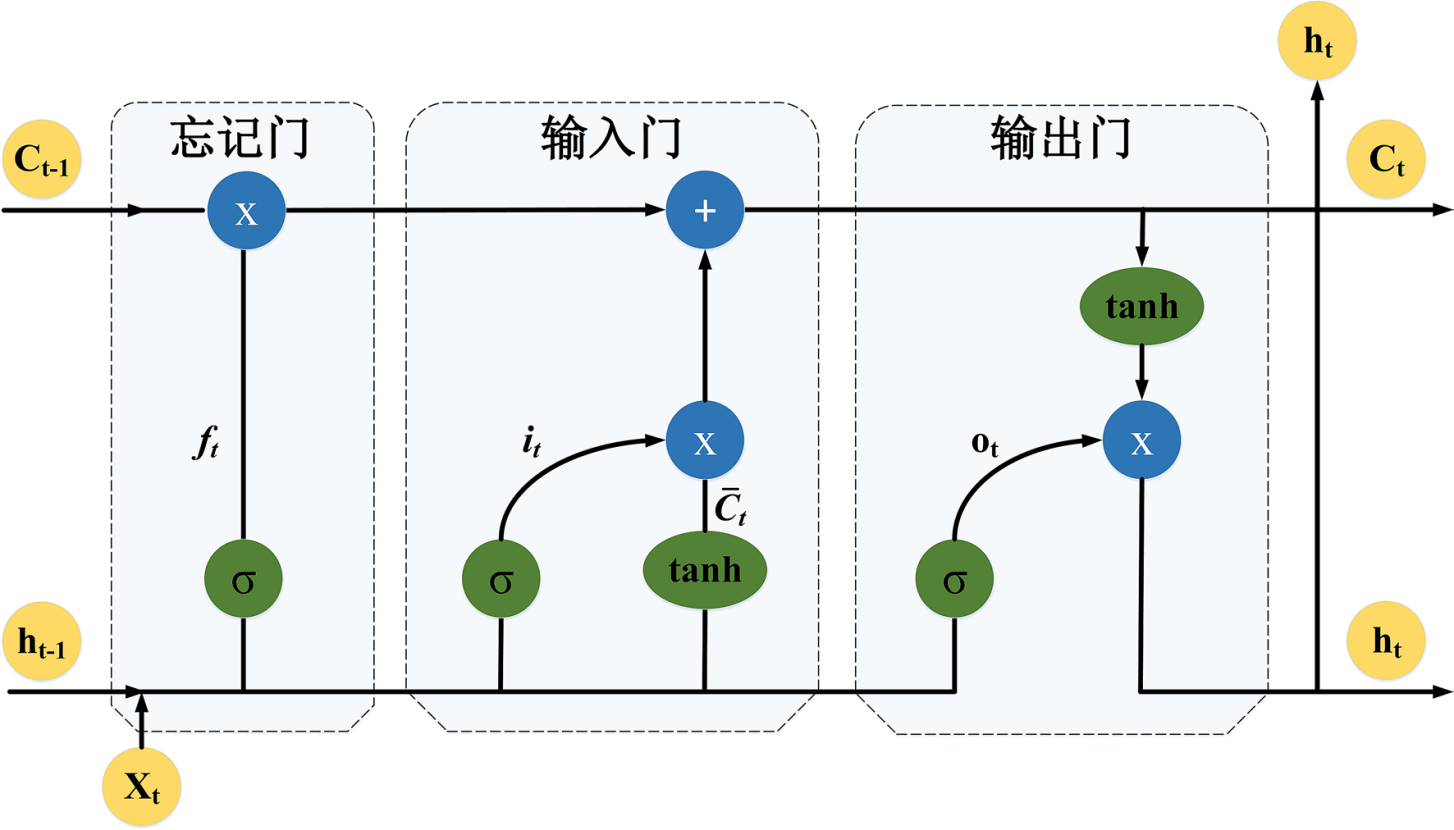
Detailed structure of a typical LSTM unit; its context and sequence learning function is based on the three gate mechanisms.

LSTM is an improvement over the RNNs, which have been previously employed in EEG analyses^33,34^. Compared to traditional RNNs, the innovation of LSTM networks is the addition of three control units (“cells”): (1) a forget gate, (2) an input gate and (3) an output gate. The structure of a typical LSTM unit is shown in Fig. 4, and the mechanisms of the gates are described as follows:

Forget gate: The gate decides what previous information should be forgotten. The current step’s input ${\mathrm{x}}_{\mathrm{t}}$ and the hidden state ${\mathrm{h}}_{\mathrm{t}-1}$ from the prior unit are concatenated into a new vector. Multiplying by the weight parameter ${\mathrm{W}}_{\mathrm{f}}$ of the gate, every element’s value of the output vector ${\mathrm{f}}_{\mathrm{t}}$ is scaled from 0 to 1 via the elementwise sigmoidal operation σ. A ‘0′ element enables the corresponding information in ${\mathrm{C}}_{\mathrm{t}-1}$ to be eliminated, while a ‘1′ means that the corresponding information is allowed to be passed through. The output ${\mathrm{f}}_{\mathrm{t}}$ of the gate is formalized as Eq. (18).

18 $$f_{t} = \sigma \left( {W_{f} \cdot \left\lceil { h_{t - 1}, x_{t}}\right\rceil + b_{f} } \right)$$

Input gate: The gate determines how much of the input ${x}_{t}$ of the network is saved to the unit state ${\mathrm{C}}_{\mathrm{t}}$. The fulfillment of the input gate’s function requires cooperation between two parallel layers. The tangent layer outputs candidate information ${\mathrm{C}}_{\mathrm{t}}$ for selection, while the sigmoidal layer acts as the forget gate and decides what candidate information will be selected by outputting the decision vector ${\mathrm{i}}_{\mathrm{t}}$. After the elementwise multiplication of the candidate information by the decision vector ${\mathrm{C}}_{\mathrm{t}}\times {\mathrm{i}}_{\mathrm{t}}$ is performed, the final update information that should be added to the cell state is determined. The function of the two layers is formalized by Eqs. (19) and (20).

19 $$i_{t} = \sigma \left( {W_{i} \cdot \left\lceil {h_{t - 1}, x_{t}}\right\rceil + b_{i} } \right)$$

20 $$\bar{C}_{t} = \tan \left( {W_{c} \cdot \left\lceil {h_{t - 1}, x_{t}}\right\rceil + b_{c} } \right)$$

Therefore, the cell state ${\mathrm{C}}_{\mathrm{t}}$ of the current chain is a combination of the reserved historical information of ${\mathrm{C}}_{\mathrm{t}-1}$, and the updating information selected from ${\mathrm{C}}_{\mathrm{t}}$ (Eq. 21).

21 $$C_{t} = C_{t - 1} \times f_{t} + \bar{C}_{t} \times { }i_{t}$$

Output gate: The gate decides which hidden state ${\mathrm{h}}_{\mathrm{t}}$ in the current chain to output via multiplication of the decision vector ${\mathrm{o}}_{\mathrm{t}}$ by the candidate information selected from ${\mathrm{C}}_{\mathrm{t}}$, as shown in Eqs. (22) and (23).

22 $$o_{t} = \sigma \left( {W_{o} \cdot \left\lceil {h_{t - 1}, x_{t}}\right\rceil + b_{o} } \right)$$

23 $$h_{t} = \tan \left( {C_{t} } \right) \times { }o_{t}$$

Our RNN, which is based on the LSTM structure, learns contextual time series information from the feature sequences extracted from the CNN and then determines the overall classification of schizophrenia or normal according to the LSTM output in each time step. Because brain activity is a dynamic process, changes between trials may contain additional information about potential mental states.

### Training and testing

We train the hybrid DNN with the optimization algorithm adaptive moment estimation (Adam)^35^, a learning factor of $1\times {10}^{-3}$ and decay rates of the first moment and second moment of 0.9 and 0.999, respectively. This work uses the Adam optimizer to update the parameters of the proposed network structure. Note that the Adam optimizer can make the network converge at a faster speed to improve the efficiency of the training process. The batch size is set to 32 to update the parameters of the proposed recurrent-convolutional network. To avoid overfitting and improve the generalization ability, dropout^36^ (set to 0.5) is applied to the FC layers. The network converges after approximately 33,660 iterations and 180 epochs.

In total, 200 training epochs were run. The epoch refers to the iteration over the entire training set. This study uses a tenfold cross-validation^37^method. First, randomly divide the EEG signals into ten equal sets. Nine groups are used as training models, and one group is used to test the system performance. By transmission testing and training of the data set, this strategy was repeated ten times. The accuracy value reported in this study is the average value obtained from ten evaluations.

### Statistical test

A statistical test was performed with SPSS 16.0. For the group comparisons of the demographic and clinical variables, we used chi-square tests for categorical variables and independent-sample *t*-tests for continuous variables. To explore the differences among the conditions, a paired *t*-test was computed. All *p* values were two-tailed, and the significance level was set to *p* < 0.05 and corrected using the false discovery rate (FDR)^38^ and Bonferroni correction ^39^. Pearson's r coefficients were computed to investigate the correlations.

### Baseline methods

Our study compared our method with various commonly employed classifiers, including the support vector machine (SVM), K-nearest neighbor (KNN) and logistics regression (LR) classifiers. All baseline methods are compared with our method using a tenfold cross-validation method. Here, we briefly describe the details and parameters used in these methods.

SVM: An SVM is based on statistical learning theory and uses kernel functions to transform linearly inseparable problems in low-dimensional space into linearly separable problems in high-dimensional space. The SVM hyperparameters, which consist of the regularization penalty parameter (C) and the radial basis function (RBF) kernel SD ($\upgamma =1/\upsigma$) inverse, are selected.

KNN: The KNN is a supervised learning algorithm that uses k-nearest examples to classify data labels. The majority vote on the sample neighbors determines the label of the sample. The Euclidean metric is used to measure distance.

LR: LR is used to describe the relationship between the independent variable X and the dependent variable Y and predict the dependent variable Y. The dependent variable Y is a real number between 0 and 1 that represents the probability of obtaining two results in the binary classification. The LR selects the optimal regularization parameter C and searches the log range of [${10}^{-2},{10}^{3}$].

## Results

### Data recording and preprocessing

The EEG data were recorded using a 64-channel EEG system produced by Brain Products, Germany, according to the international 10–20 system. The impedances were kept below 5 kΩ, and the sampling rate was 500 Hz. During recording, each participant was seated upright and asked to remain quiet and relaxed with open eyes in an acoustically and electrically shielded room. In this experiment, the recording time lasted 2 min. In addition, the effects of brain activity and psychological factors were disregarded.

The whole data preprocessing and analysis procedure was implemented on BrainVision Analyzer 2.0. First, new referencing was used to select the available electrodes (The following electrodes were deleted: HEOGL, HEOGR, VEOGL, and VEOGU.). Second, bandpass filtering was used to obtain the frequency bands from 0.5 to 50 Hz with a slope of 24 dB/oct. The EEG recordings were divided into 1,400-ms lengths for each segmentation and 55 trials. Third, baseline correction was performed in the range for the mean value calculation beginning at 0 ms and ending at 1400 ms to eliminate EEG noise caused by spontaneous EEG activity. Fourth, ocular correction was utilized to correct the signal interference caused by blinking or eye movements. Artifact rejection was then used to remove the false signals produced by the equipment or the action of the subject. Last, the EEG data of the 60 electrodes were exported for further analysis.

### Comparison of the feature

**Figure 5:**
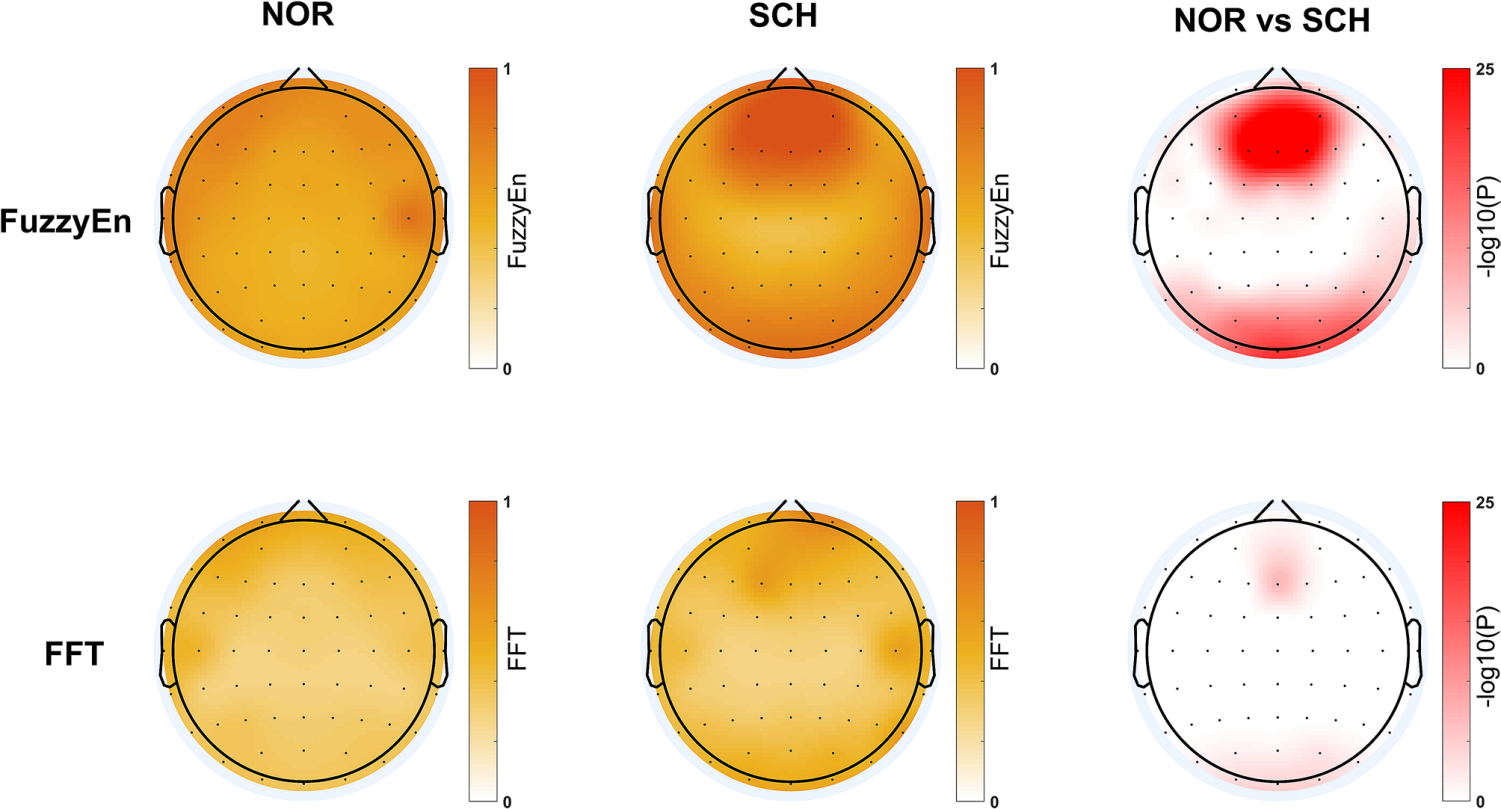
Brain topographic maps for different features of the two groups. In the first two columns, the darker the color is, the higher the feature values are. The third column takes the logarithm of the p-value of the statistical test: the redder the color is, the greater the differences are.

For the group analysis, a relation analysis based on the direct contrast between the schizophrenia patients and the healthy controls was carried out to generate the brain topographic map for each group (significance level = 0.05). As shown in Fig. 5, brain topographic maps of the FuzzyEn and FFT features are displayed for the entire time series.

The FuzzyEn maps show that the FuzzyEn value of the patients with schizophrenia is significantly larger than that of the normal controls (corrected *p* < 0.05), and large differences exist in the frontal region compared with other regions. The FFT maps show that the FFT values for the schizophrenia patients are similar to those for the normal control group (corrected *p* < 0.05), and no significant differences exist between the regions of the brain from the FFT. In addition, we applied a repeated-measures analysis of variance (ANOVA) as the statistical tool. Depending on the ANOVA results, we evaluated the statistical significance of the differences in the features between the groups of subjects and observed a significant group effect [F = 334.208; *p *< 0.001] in FuzzyEn and a small group effect [F = 9.595; *p * < 0.01] in FFT.

### Classification results

We present our classification results derived by extracting the FuzzyEn and FFT features from the 6 time windows. The purpose was to seek the best-performing models for the generated images. The accuracies for the validation set and the test set of our proposed methods and baseline methods are reported in Table 5.

**Table 5:** Comparison of the classification results.

Method	Model	FuzzyEn		FFT	
		Validation accuracy (%)	Testing accuracy (%)	Validation accuracy (%)	Testing accuracy (%)
Baseline methods	SVM	**–**	93.01	**–**	88.97
	KNN	**–**	91.72	**–**	91.70
	Logistic Regression	**–**	91.38	**–**	91.36
Proposed methods	A < 2,0,0 >	**–**	92.33	**–**	90.12
	B < 2,2,0 >	98.66	96.34	97.50	93.07
	C < 2,2,1 >	99.22	**99.22**	98.44	96.34
	D < 4,2,1 >	99.34	94.94	98.44	92.49

We discovered that the accuracy of our proposed methods for the validation set is higher than that for the test set because the test set was selected from the model with the highest accuracy for the validation set. Considering the randomness of the data, the models with the highest accuracy for the validation set may not be those with the highest accuracy for the test set, so the calculated indicators from the validation set are generally better than those from the test set.

We determine that the accuracy of the FuzzyEn feature is higher than that of the FFT feature. For the baseline methods, the FuzzyEn value of each model is higher than the FFT value in terms of the testing accuracy. For the proposed methods, the FuzzyEn value of each model is higher than the FFT value in terms of the testing accuracy and the verification accuracy. Therefore, we can conclude that the FuzzyEn features have a more substantial role in the classification results than the FFT features.

In the proposed methods, we discover that the testing accuracy is different when the convolution layer is different. The findings negate the notion that the greater is the number of layers that are included, the higher is the accuracy. In particular, using FuzzyEn features, we achieve the best performance with the B architecture (testing accuracy of 99.22%), which contains 5 convolution layers and is marginally better than the other methods.

**Figure 6:**
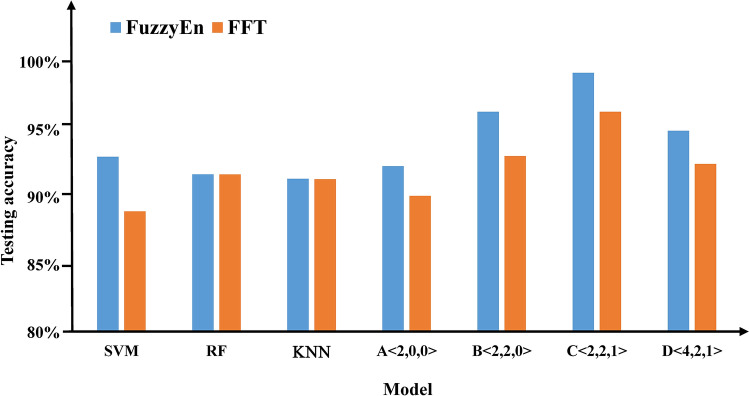
Testing accuracies of the two characteristics for all of the methods. The x-axis represents the different classifiers, and the y-axis represents the testing accuracy. The blue column represents the FuzzyEn value, and the orange column represents the FFT value.

Figure 6 shows a bar graph of the testing accuracies of the two features for all the methods. We observe that the proposed methods are superior to the baseline methods. We determined that the best result is obtained with the C < 2,2,1 > architecture and the FuzzyEn feature. The differences among the accuracy rates between the four DL methods are not statistically significant.

**Figure 7:**
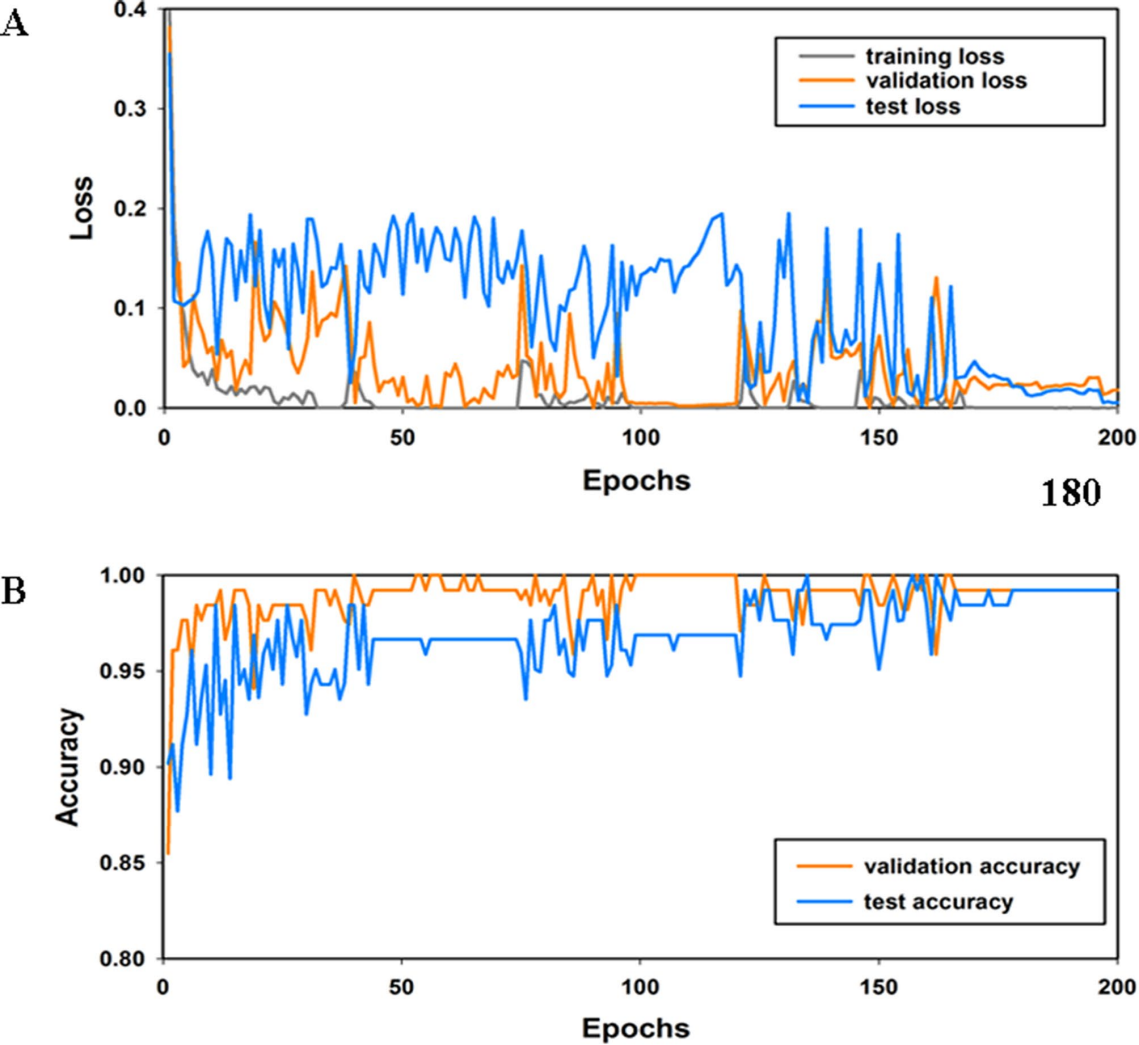
Loss curve and accuracy curve of the best architecture. (**A**) Shows the loss curve of the best architecture. The blue curve represents the testing loss, the orange curve represents the validation accuracy, and the gray curve represents the training loss. (**B**) Represents the accuracy curve of the best architecture. The blue curve represents the testing accuracy, and the orange curve represents the validation accuracy.

To further study the performance of the best-performing architecture (i.e., C architecture), we draw the accuracy and loss curves in Fig. 7. From the accuracy curve in Fig. 7A, we can observe that as the number of training epochs increases, the validation and testing accuracy show an overall upward trend. When the number of epochs of training reaches 180, the algorithm quickly converges to an ideal state, and the accuracy tends to be stable and reaches approximately 99.22%. In contrast to the accuracy curve, Fig. 7B shows that the training loss, validation loss and test loss are gradually reduced, and when the number of training epochs reaches 180, the testing loss reaches a stationary state of approximately 0.019.

Previous studies have shown that the performance of the trained model is significant for both the validation set and the test set and can be simultaneously judged by the training loss and the validation loss and reaches the stable state; thus, the base model that was trained in this experiment shows a satisfactory fit.

## Comparison with different deep learning models

To quantify the importance of the 6 time windows for our results, we also applied 5 time windows and 7 time windows and retrained our network. The results show that the classification effect of using 6 time windows is better than the classification effects achieved with the other time windows for both the DL methods and the ML methods.

Table 6 compares the training times and the number of parameters among the proposed methods. We note that the more complex is the structure of the network, the longer is the training time and the higher is the number of parameters. Most network parameters are located in the last two layers (i.e., FC layer and SF layer) and require more storage and computing power during training and testing. From the obtained results, the accuracy of the C model is the highest, and the training time and number of training parameters are moderate. Therefore, we focus on the C model in the following discussion.

**Table 6:** Comparison of the training times and number of parameters of different structures.

Model	Train time (per epoch)	Number of parameters
A < 2,0,0 >	1185.596 s	557,885
B < 2,2,0 >	2209.655 s	906,717
C < 2,2,1 >	2696.328 s	1,806,749
D < 4,2,1 >	4276.018 s	1,825,565

In the VGG style network, to keep the size of the output after each stack constant (filter size × number of cores), we determined the number of filters in each layer. In addition, we manually reduced the amount of data that is required to manually extract power features from EEG signals.

## Discussion

### Comparison of the FuzzyEn feature and FFT feature

EEG signals are complex nonlinear dynamic signals^5^ and contain the dynamic properties of brain activity^40^. In this study, the eigenvalues of schizophrenic patients were higher than those of healthy controls. This finding is consistent with previous results. Fernández A et al. employed the Lempel–Ziv complexity (LZC) method to study healthy controls and patients with schizophrenia and discovered that in terms of time-domain characteristics, patients show higher performance in the entire brain complexity ^41^. The EEG signals of subjects with schizophrenia were more random, and therefore, had a greater approximate entropy compared to the EEG signals of healthy subjects^42^. In addition, as previously reported using multiscale entropy, the complexity of schizophrenic patients is higher than that of the control group^43^. In the frequency-domain features, patients with schizophrenia had significantly higher theta power over the F4, F7, F8, P4 and O2 regions than healthy subjects^44^. Compared with the healthy control group, schizophrenic patients have more active brain activity and are more likely to generate new EEG signal patterns^45^.

The statistical test results show that both the FuzzyEn feature and the FFT feature can be used to distinguish healthy controls from patients with schizophrenia, but the FuzzyEn feature is more significant and more easily distinguished between healthy controls and patients with schizophrenia. The main differences between schizophrenic patients and healthy controls are concentrated in the frontal regions. This is consistent with previous research findings^46–49^. The frontal area is mainly responsible for memory problems related to behavior regulation and cognitive perception ^50^. The impairment of metacognitive function in patients with schizophrenia may be caused by the frontal region^51^. According to previous research on social cognition in schizophrenia^52^, the abnormality observed in this area is based on dopamine signaling to the prefrontal cortex.

In addition to the differences in brain topographic maps, the two features are also different in terms of the classification performance. For the classification results, the FuzzyEn feature performs better than the FFT feature in all methods. The EEG signals using FuzzyEn features are more effective for classification than those using FFT features. For these results, the FuzzyEn feature retains the time sequence, which adequately remembers the relative characteristics of the original signals during the sliding of the time window. The hybrid DNN, which is based on FuzzyEn features, combines the time-, frequency- and spatial-domain characteristics. Therefore, the hybrid DNN may more easily extract additional effective features based on the FuzzyEn feature than the FFT feature.

### Comparison with other studies of schizophrenia

The classification problem of schizophrenia EEG signals involves extracting the discriminative features from EEG signals and then performing the classification. We compare our research with the related state-of-the-art techniques that have been developed in recent years, as shown in Table 1, which use different feature extraction and classification methods for classifying schizophrenia EEG signals.

In 2016, Bose et al. developed an SVM filter for the identification of schizophrenia based on the delta, theta, alpha, and beta bands that were extracted from EEG signals using a finite impulse response bandpass filter. In alpha power, the subject groups yielded a high classification accuracy of 83.33%. These results suggest that schizophrenic subjects can be identified by the absolute alpha ^16^. Johannesen et al. employed a Morlet continuous wavelet transform to extract time–frequency features in healthy communities and schizophrenic patients from EEG signals, and the SVM provided the highest classification accuracy of 87%^53^.

In 2017, Jeong et al. developed a multimodal (audiovisual) emotion perception test. The discriminatory features were extracted using a mean subsampling technique from EEG recordings. Shrinkage linear discriminant analysis (SKLDA) can decrease the ill-conditioned covariance matrix, which provides a more accurate classification of the event-related potential (ERP), even when using an insufficient training sample size. Thus, SKLDA was employed for the classification and attained more than 98% accuracy^54^. Piryatinska et al. created a low-dimensional feature space, which decomposed the EEG signals of adolescent schizophrenic and control subjects using the є-complexity of a continuous vector function. They utilized a random forest (RF) classifier and achieved an average accuracy of 85.3%^55^. Chu et al. used three different types of International Affective Picture System (IAPS) pictures as visual stimuli and captured the associated brainwaves. They then employed the approximate entropy (ApEn) to extract features and classified them with an SVM. The researchers discovered that the classification accuracy of healthy controls and schizophrenic patients with obvious illness was as high as 81.5%^56^.

The following year, Alimardani et al. proposed an efficient feature selection algorithm named Davies–Bouldin fast feature reduction (DB-FFR) to select the most discriminative features to enhance the classification rate. These researchers applied a modified version of the KNN classifier and achieved an 87.51% classification accuracy for the EEG features of schizophrenia patients and bipolar mood disorder (BMD) patients^57^. The researchers also applied the steady-state visual evoked potential (SSVEP) of the EEG signals and extracted the power spectral densities. The feature was fed into five classifiers to characterize the EEG signals, and the KNN classifier provided the highest classification accuracy (91.30%), with the best feature set selected by the Fisher score between BMD and schizophrenic patients^58^.

In 2019, Phang et al. proposed a DNN with a deep belief network (DBN) architecture for the automated classification of schizophrenia (SZ) based on the EEG effective connectivity. The structure has a multilayer architecture as an inherent feature extractor, which is able to learn hidden hierarchical representations of the complex brain network structure. These researchers employed directed connectivity (DC) based on vector autoregression (VAR), graph theory composite network (CN) metrics, and a combination of both as input features, and achieved 95% significant classification accuracy for the θ and β bands^59^. In addition, for the same subjects, they applied combinations of various connectivity features as input features, including time- and frequency-domain metrics of the effective connectivity based on the VAR model and partial directed coherence (PDC), with complex network (CN) measures of network topology. The researchers designed a novel multidomain connectome CNN (MDC-CNN) based on a parallel ensemble of one dimensional (1D) and 2D CNNs to integrate the features from various domains and dimensions using different fusion strategies. The results showed that the MDC-CNN with combined connectivity features further improved the performance over single-domain CNNs and achieved a remarkable accuracy of 91.69% with a decision-level fusion^60^. In the same year, Oh et al. established an eleven-layer CNN model to directly process the original EEG signals for analysis without any feature processing. The proposed model generated a classification accuracy of 98.07% using nonsample testing^14^.

In the comparative studies, the researchers collected the subjects themselves. Our subjects were in accordance with the usual format of the current collection: resting data for patients with small requirements, sampling rate = 500, and channels = 60. In Table 1, we also add these conditions. After observation, a unified conclusion cannot be obtained. In different studies, different information is collected for the subjects, different features are extracted, and different classifiers will affect the accuracy. Because of data limitations, the impact of the model is unknown. Given that a long time is needed to collect new data, we did not investigate it but will explore it in future studies. Therefore, we mainly focus on the accuracy of this study to show that the distinction between normal and schizophrenic patients can be achieved with a high accuracy.

It can be seen from Table 1 that most previous studies applied machine learning techniques to diagnose schizophrenia. These traditional methods are cumbersome and require feature extraction and selection before classification. In addition, these methods perform poorly when using large data sets. In some recently applied DL methods, the classification results of these studies have greatly improved compared with machine learning methods.

In this study, we propose a hybrid deep neural network model to classify schizophrenia. Compared with previous research, our experimental data are more numerous, and the amount of data is enlarged by sliding the time window. Before entering the model, we retained the brain electrodes by AEP mapping. This method has not appeared in previous research. We selected fuzzy entropy features and constructed the hybrid DNN model, which combines the advantages of a CNN and an LSTM. The classification accuracy rate reached 99.22%, and the experimental results are superior to those of previous studies. This result shows that our method provides a significant breakthrough in the classification of schizophrenia based on EEG data. In future research, the hybrid deep neural network classification method can also be applied to the diagnosis of EEG signals and other classifications of medical diseases.

## Conclusions

In this study, we attempted to compare which feature of FuzzyEn and FFT is better and improve the accuracy of classification of schizophrenia in EEG signals. Our method involves two procedures. First, we convert EEG signals into a series of topology-preserving RGB images rather than standard EEG analysis techniques that disregard this spatial information. Second, we use a hybrid DNN that consists of CNN and LSTM components to address the RGB images and differentiate schizophrenic patients and healthy controls. In the hybrid structure, the CNN is used to process the RGB images and extract features from them, and an LSTM is used to structure the contextual information for long-term sequences of arbitrary length.

We compared the features of FuzzyEn and FFT and discovered that the FuzzyEn feature has a better effect than the FFT feature in terms of the classification accuracy. In addition, we determined that the maximum classification accuracy of our proposed method can reach 99.22%, which is higher than the accuracy of the baseline method. In addition, we review the latest methods of schizophrenia classification based on EEG signals. Compared with these studies, our study significantly improves the classification accuracy.

In the future, the proposed method will be trained using a more powerful graphics processing unit (GPU) to optimize the training time. A larger cohort of subjects with schizophrenia/healthy controls will be taken into account to further demonstrate and fully exploit the generalization potential of DL techniques for clinical applications.
